# *Terminalia chebula* extract prevents scopolamine-induced amnesia via cholinergic modulation and anti-oxidative effects in mice

**DOI:** 10.1186/s12906-018-2212-y

**Published:** 2018-05-02

**Authors:** Min-Soo Kim, Dong Young Lee, Jun Lee, Hyun Woo Kim, Sang Hyun Sung, Jung-Soo Han, Won Kyung Jeon

**Affiliations:** 1 Herbal Medicine Research Division, Korea Institute of Oriental Medicine, Daejeon, 34054 South Korea; 2 Convergence Research Center for Diagnosis, Treatment and Care System of Dementia, Korea Institute of Science and Technology, Seoul, 02792 South Korea; 3 College of Pharmacy, Seoul National University, Seoul, 08826 South Korea; 4 Department of Biological Sciences, Konkuk University, Seoul, 05029 South Korea

**Keywords:** *Terminalia chebula*, Amnesia, Cholinergic system, Oxidative damage

## Abstract

**Background:**

*Terminalia chebula* Retz. (Combretaceae) is a traditional herbal medicine that is widely used in the treatment of diabetes, immunodeficiency diseases, and stomach ulcer in Asia. However, the anti-amnesic effect of *T. chebula* has not yet been investigated. The present study was designed to determine whether *T. chebula* extract (TCE) alleviates amnesia induced by scopolamine in mice. We also investigated possible mechanisms associated with cholinergic system and anti-oxidant effects.

**Methods:**

TCE (100 or 200 mg/kg) was orally administered to mice for fourteen days (days 1–14), and scopolamine was intraperitoneally injected to induce memory impairment for seven days (days 8–14). Learning and memory status were evaluated using the Morris water maze. Hippocampal levels of acetylcholine (ACh), acetylcholinesterase (AChE) and choline acetyltransferase (ChAT) were measured ex vivo. Levels of reactive oxygen species (ROS), nitric oxide (NO), and malondialdehyde (MDA) in the hippocampus were also examined.

**Results:**

In the Morris water maze task, TCE treatment reversed scopolamine-induced learning and memory deficits in acquisition and retention. TCE reduced hippocampal AChE activities and increased ChAT and ACh levels in the scopolamine-induced model. Moreover, TCE treatment suppressed scopolamine-induced oxidative damage by ameliorating the increased levels of ROS, NO, and MDA.

**Conclusion:**

These findings suggest that TCE exerts potent anti-amnesic effects via cholinergic modulation and anti-oxidant activity, thus providing evidence for its potential as a cognitive enhancer for amnesia.

**Electronic supplementary material:**

The online version of this article (10.1186/s12906-018-2212-y) contains supplementary material, which is available to authorized users.

## Background

As many countries are transforming into aging societies, an increasing number of individuals in elderly populations suffers from memory loss and amnesia: worldwide cost of care and medicine for amnesia was reported to be $604 billion in 2010 and has been increasing annually [1]. The prevention or treatment of amnesia is thus an urgent issue to address.

The hippocampus and cortex are heavily involved in maintaining and regulating memory; changes in levels of the neurotransmitter acetylcholine (ACh), which is released via cholinergic projections to these areas from the basal forebrain, have been reported to affect cognitive function and have been implicated in memory loss [2]. Therapeutic interventions aiming to alleviate cognitive impairments have thus targeted ACh regulation and degradation; acetylcholinesterase (AChE) inhibitors blocking ACh hydrolysis have been administered to treat amnesia [3] and are widely prescribed to alleviate general cognitive decline [4]. However, these drugs have short half-lives and adverse reactions, including hepatotoxicity and nausea [5]. By contrast, medicinal herbs and plants exhibit fewer side effects and drug interactions [6]; identifying effective alternative medicines could thus yield valuable contributions to the treatment of amnesia.

*Terminalia chebula* Retz. (Combretaceae) has been used as a traditional medicine to treat diabetes, immunodeficiency diseases, and stomach ulcers in across Asia [7–9]. These medicinal extracts contain terpenes, flavonoids, and alkaloids, all of which exhibit therapeutic efficacy [10]. Several in vitro studies have further reported that *Terminalia chebula* extract (TCE) has anti-AChE and anti-oxidative effects: TCE has been shown to inhibit H_2_O_2_-induced PC12 cell death [11], and its methanolic and ethyl acetate fractions have demon strated varying degrees of AChE inhibitory activity [12, 13]. Despite the progress made to explore the therapeutic properties of TCE, whether the extract features an anti-amnesic effect has not yet been investigated.

Scopolamine is a muscarinic ACh receptor antagonist that can cause learning and memory deficits by disrupting cholinergic neurotransmission; this compound has been used to induce amnesia in experimental murine models [14]. In the present study, we evaluated the memory-enhancing effects of TCE on scopolamine-treated mice using the Morris water maze test. We subsequently investigated the levels of ACh, AChE, and choline acetyltransferase (ChAT) in the hippocampus. To elucidate any anti-oxidative activities of TCE, we also ascertained the effects of TCE on reactive oxygen species (ROS), nitric oxide (NO), and malondialdehyde (MDA) in mice hippocampal tissue.

## Methods

### Reagents

Scopolamine hydrobromide, sodium nitrite (NaNO2), Griess reagent, dimethyl sulfoxide (DMSO), dichlorofluorescin diacetate (DCFDA), phosphate buffer, DPPH, donepezil, and ascorbic acid were purchased from Sigma-Aldrich (St. Louis, MO, USA). Anti-ChAT and glyceraldehyde 3-phosphate dehydrogenase (GAPDH) were purchased from EMD Millipore (Billerica, MA, USA). Anti-AChE was obtained from Abcam (Cambridge, MA, USA). Radioimmunoprecipitation assay (RIPA) buffer was obtained from Thermo Scientific (Waltham, MA, USA). All horseradish peroxidase-conjugated secondary antibodies were purchased from Santa Cruz (Santa Cruz, CA, USA). Chebulic acid, gallic acid, corilagin, chebulanin, 1,3,6-tri-O-galloyl β-D-glucose, chebulagic acid, and chebulinic acid were provided by Professor Sang Hyun Sung (Seoul National University, College of Pharmacy, South Korea). The purity of all reference chemical substances was higher than 95%. HPLC-grade water and acetonitrile were purchased from J.T. Baker (Phillipsburg, NJ, USA).

### Plant materials and extraction

*Terminalia chebula* Retz. is an accepted plant name and is listed in The Plant List (www.theplantlist.org). The ripe fruit of *Terminalia chebula* were purchased from Kwangmyungdang Medicinal Herbs Co. (Ulsan, South Korea) and identified by Dr. Go Ya Choi from the K-herb Research Center (Korea Institute of Oriental Medicine, Daejeon, South Korea). A voucher specimen (KIOM-Tech5) was deposited at the Convergence Research Center for Diagnosis, Treatment and Care System of Dementia (Korea Institute of Science and Technology, Seoul, South Korea).

The ripe fruit of *Terminalia chebula* (1.0 kg) were ground and soaked in 70% EtOH (10 L for 15 h) at 40 °C. The 70% EtOH extracts were filtered, concentrated (40 °C, EYELA rotary evaporation system, Tokyo Rikakikai, Tokyo, Japan), and dried (PVTFD-100 freeze drier, Ilshinbiobase, Dongducheon, South Korea) to produce a powdered extract (268.4 g), which was stored at − 20 °C.

### Animals

A total of 56 eight-week-old male C57BL/6 N mice were obtained from Charles River Co (Gapyeong, South Korea). We housed four to five animals per cage at a controlled temperature (23 ± 2 °C) and humidity (50 ± 10%). The mice were kept on 12 h/12 h light/dark cycle (light from 08:00 A.M.- 08:00 P.M.). The mice received food and water ad libitum. The institutional animal care and use committee of Korea Institute of Science and Technology approved all of the experimental protocols described in the present study (Permit number: 2016–068). All procedures for the animal study were conducted in accordance with ARRIVE guidelines, and every effort was made to alleviate the suffering of the animals.

### Pharmacological treatment

After adaptation for one week, the mice were randomly divided into six groups: (1) vehicle + vehicle (*n* = 10); (2) TCE (200 mg/kg) + vehicle (TCE per se; *n* = 6); (3) vehicle + scopolamine (scopolamine per se; *n* = 10); (4) TCE (100 mg/kg) + scopolamine (*n* = 10); (5) TCE (200 mg/kg) + scopolamine (*n* = 10); and (6) donepezil (5 mg/kg) + scopolamine (*n* = 10). The doses of TCE were determined based on previous report [15]. We used saline for the vehicle group; TCE was therefore suspended in saline. The mice were orally administered saline, donepezil, or TCE on days 1–7 to adapt them to the oral gavage and to adapt their metabolism prior to behavior tasks; treatment continued on days 8–14. Scopolamine (1 mg/kg) was dissolved in saline and treated intraperitoneally for 7 days (days 8–14). On days 8–14, TCE and scopolamine were administered at 60 min and 30 min before training, respectively.

### Morris water maze task

Spatial learning and memory was evaluated using the water maze task as described in a previous study with minor modifications [16]. All mice underwent behavioral testing 30 min after scopolamine injection. The water maze comprised of a white circular tank (183 cm diameter and 58 cm height) with a circular platform (20 cm diameter). The tank was filled with water (25 ± 1 °C) and a nontoxic white dye. The platform hidden 0.5 cm beneath the water surface in one of tank’s quadrants. The maze was enveloped in white curtains on which black patterns were placed as visual cues. All data were monitored using a camera recorder and analyzed using a video tracking system (HVS Image, Hampton, UK).

On days 8–13, the mice underwent four training trials. If the mouse found the escape platform within 60 s, the animal was left on the platform for an additional 20 s. If the mouse did not find the platform within 60 s, the animal was placed on the platform and permitted to remain there for 20 s. The starting positions of the mice were changed across trials. Search errors were used as a metric for performance accuracy during training trials: the distance of the mouse from the platform was sampled 10 times per second, averaged across 1 s, and its deviation from the most direct search path was calculated [17]; this method allowed us to remove a time bias associated with the repositioning of the platform. On days 11 and 13, all mice were subjected to probe tests without a platform for a 30 min interval between the last training trial and the probe trial to examine the retention of spatial memory. The average swimming speed of each mouse and their time spent in the target quadrant was measured.

### Sacrifice of mice

**Fig. 1:**
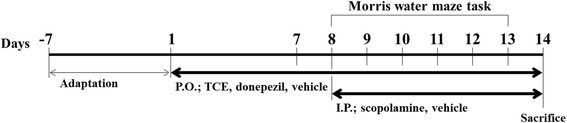
Schematic description of the experimental design. After a 1-week adaptation, C57BL/6 N mice were orally treated with vehicle (saline), TCE (100 or 200 mg/kg), or donepezil (5 mg/kg) for 14 days. From day 8, the mice were also treated intraperitoneally with vehicle (saline) or scopolamine (1 mg/kg) for 7 days. The Morris water maze was conducted on days 8–13 to evaluate memory function. All mice were sacrificed on day 14. The hippocampus was then collected for further biochemical analyses. P.O.; per os (by mouth, orally), I.P.; intraperitoneal

On day 14, all mice were sacrificed 30 min after injection of scopolamine or the vehicle; cervical dislocation was performed before decapitation to provide each animal with a quick and painless death. The hippocampus was placed on ice and stored at − 80 °C until further biochemical analyses. All the mice were handled according to the animal welfare guidelines issued by Korean National Institute of Health. The experimental scheme is depicted in Fig. 1.

### Determination of ex vivo ACh and AChE activity

The activities of hippocampal ACh and AChE were determined using commercially available ACh quantitation and AChE activity assay kits according to the manufacturer’s instructions (Sigma). The absorbance of ACh and AChE were measured using a microplate reader at 570 nm and 412 nm, respectively (Bio Tek, Winooski, VT, USA). The values were reported as percentages of the vehicle control (vehicle + vehicle).

### Western blot analysis

Sample preparation and western blotting were performed according to a previous report with minor modifications [16]. The tissues were homogenized in an RIPA buffer containing protease and phosphatase inhibitors (GenDEPOT, Barker, TX, USA). They were then centrifuged at 14,000 × g for 1 h at 4 °C. The supernatants were collected, and Bradford assays were performed to determine protein concentrations. The proteins (30 μg) were separated using SDS-PAGE and subsequently transferred to PVDF membranes. After 1 h of incubation in 5% fat-free dry milk, the membranes were incubated overnight with primary antibodies against ChAT (1:1000), AChE (1:1000), and GAPDH (1:5000) at 4 °C. The bands were normalized to GAPDH and quantified using an Image Gauge program (Fujifilm, Tokyo, Japan).

### Determination of ROS and NO levels

ROS levels were determined according to a previous report [18]. Hippocampal homogenates were incubated with 15 μl of 1 mM DCFDA in the dark at 37 °C for 1 h. The fluorescence intensity was measured using a fluorescence microplate reader (Spectramax M5, Molecular Devices, Pennsylvania, USA) with an excitation and emission of 488 and 520 nm, respectively. The values were reported as a percentage of the vehicle control (vehicle + vehicle).

NO production in the hippocampus was measured using the Griess method [19]. We incubated 40 μl of the sample with 160 μl of the Griess reagent in the dark at room temperature for 15 min. NaNO_2_ was used to generate a standard nitrite concentration curve. The purple Azo dye product was detected at a wavelength of 540 nm. The amount of NO was expressed as μmole per mg protein.

### Estimation of lipid peroxidation

MDA has been widely used to assess lipid peroxidation. The MDA concentration in the hippocampus was estimated using a commercial lipid peroxidation (MDA) assay kit according to the manufacturer’s instructions (Abcam). Its absorbance was measured using a microplate reader at 532 nm. The concentration of MDA was calculated using a reference standard. The results were expressed as nmole per mg protein.

### High-performance liquid chromatography (HPLC) analyses

HPLC analysis was performed using a Thermo Scientific Dionex Ultimate 3000 system. TCE and a mixed standard solution were separated using an YMC Triart C18 column (4.6 mm × 250 mm, 5.0 μm); the temperature of the column was maintained at 30 °C during the process. The mobile phases comprised of 0.1% (*v*/v) formic acid in water (solvent A) and acetonitrile (solvent B). The following gradient system was used: (B) = 3%–30% (0–30 min) and 30–90% (30–35 min). System re-equilibration lasted 10 min. Analysis was performed at a flow rate of 1.0 mL/min with UV detection at 280 nm. The injection volume was 2 μl. All analyses were made in triplicate.

### Statistical analysis

All data were expressed as the means ± standard error of the mean (S.E.M). Statistical analysis was performed using SPSS software (SPSS Inc., Chicago, IL, USA). The search error data from the behavioral test were analyzed using two-way repeated analysis of variance (ANOVA). The correlation between obtained data was analyzed by the Pearson test. Other data were analyzed using one-way ANOVA. Post hoc analyses were subsequently performed using the least significant difference (LSD) test for comparison among experimental groups. The statistical significance was defined as *p* values of less than 0.05.

## Results

### Effect of TCE on learning and memory improvement in the Morris water maze

All experimental groups showed no toxicity in terms of general behavioral changes or mortality, and no adverse events were observed. Mouse body weights were measured on days 1 through 14 during treatment with TCE, donepezil, and scopolamine; no significant differences were found among the different groups (data not shown).

**Fig. 2:**
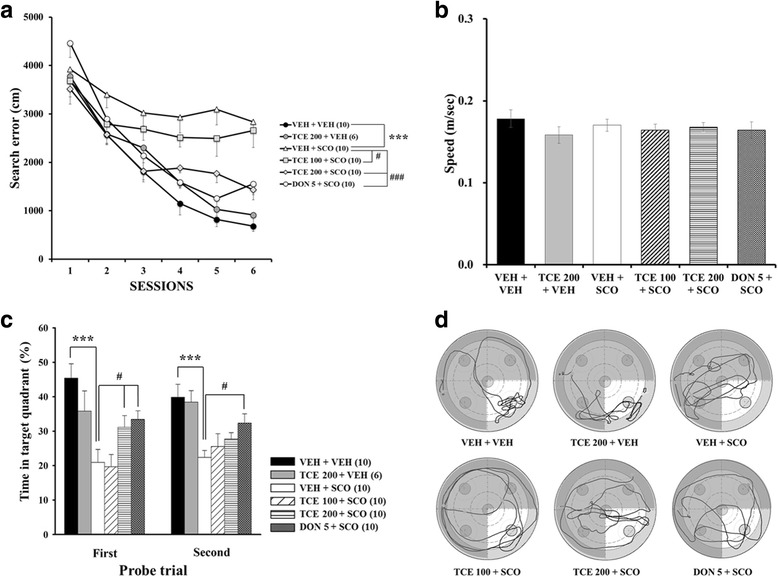
Effect of TCE on scopolamine- induced memory impairment in the Morris water maze task**. a** Acquisition of spatial memory in mice. The cumulative search error (distance from the mice to escape platform) during 6 days of training is shown. The number in parentheses indicates the size per group (*n*). **b** Mean swimming speed of each group on the first day of training. **c** Retention of spatial memory in mice. Time spent in the target quadrant during the probe trial on day 11 (1st) and 13 (2nd) is shown. **d** Representative swimming tracks of mice in each group during the 1st probe test. The bright part indicates the target quadrant. The data are expressed as the means ± S.E.M. ***p < 0.001 compared with VEH + VEH; #*p* < 0.05, ###*p* < 0.001 compared with VEH + SCO. VEH; vehicle, SCO; scopolamine, DON; donepezil

The Morris water maze test was performed to determine whether TCE attenuates learning and memory impairment induced by scopolamine. Two-way repeated ANOVA for search error revealed significant between-group treatment effects (F_(5,50)_ = 15.468, *p* < 0.001), training effects (F_(5,250)_ = 78.640, *p* < 0.001), and treatment × training interaction effects (F_(25250)_ = 3.310, *p* < 0.001). As shown in Fig. 2a, scopolamine per se treatment showed a higher search error than VEH + VEH control during the 6 days of training; this result suggests that scopolamine triggered learning deficits (*p* < 0.001). However, the scopolamine-treated mice that received either 100 or 200 mg/kg of TCE performed significantly better than those that received scopolamine alone, indicating that learning impairment was attenuated following TCE treatment (*p* < 0.05 and *p* < 0.001, respectively). Mice treated with donepezil (5 mg/kg), a well-known reversible AChE inhibitor used as a positive control, showed better performance than mice injected with scopolamine alone (*p* < 0.001). There was no difference in TCE per se-treated mice compared to vehicle-treated mice. In addition, the swimming speed did not differ among the groups, indicating that the locomotor activity of mice was not affected by scopolamine, TCE, or donepezil (Fig. 2b, F_(5,50)_ = 0.766, *p* = 0.578). We also measured the body weights of all mice during drug treatment and behavioral tasks, but there was no significant difference among the six groups (data not shown).

The mice underwent two probe trials to determine memory retention on days 11 (1st probe) and 13 (2nd probe). In the 1st probe test, one-way ANOVA revealed significant differences between-group effects (F_(5,50)_ = 9.568, *p* < 0.001). Post hoc analyses showed that the time to the target quadrant of the scopolamine per se-treated group was significantly lower than that of the vehicle control (Fig. 2c; *p* < 0.001). Treatment with TCE (200 mg/kg), however, significantly increased the time to the target quadrant (*p* < 0.05). Donepezil showed effects similar to those of TCE (200 mg/kg) treatment (*p* < 0.05). In addition, there was no difference between the TCE per se-treated mice and the vehicle control.

In the 2nd probe test, one-way ANOVA revealed significant between-group effects (F_(5,50)_ = 5.411, *p* < 0.001). Similar to the results from the 1st probe, there was a significant difference between the VEH + VEH and VEH + SCO groups (*p* < 0.001). Though TCE-treated mice spent more time in the target quadrant than scopolamine per se group, the effect was nonsignificant. The swimming routes further confirmed that TCE-treated mice remained in the target quadrant longer than did the scopolamine per se treated mice (Fig. 2d). Taken together, these results suggest that TCE ameliorated induced cognitive impairments related to spatial learning and memory.

### Effect of TCE on cholinergic system in the hippocampus

**Fig. 3:**
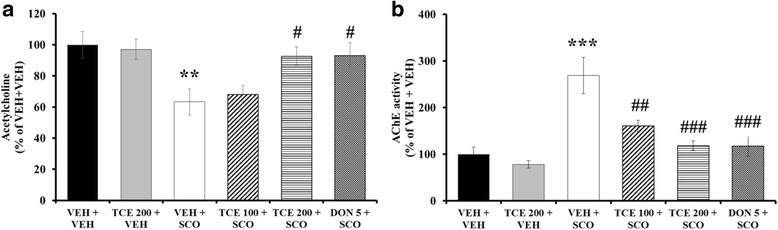
Effect of TCE on hippocampal ACh levels and AChE activity in the scopolamine-induced model. **a** The level of ACh in hippocampal tissue is shown (*n* = 3 per group). **b** Hippocampal AChE levels are presented (*n* = 5 per group). Bar graph is expressed as a percentage of VEH + VEH control. The data are expressed as the means ± S.E.M. *p < 0.05, ***p < 0.001 compared with VEH + VEH; #*p* < 0.05, ##*p* < 0.01, ###*p* < 0.001 compared with VEH + SCO. VEH; vehicle, SCO; scopolamine, DON; donepezil

To investigate whether TCE administration had an effect on cholinergic pathways, ACh levels in the hippocampus was measured. One-way ANOVA indicated significant between-group effects (F_(5,17)_ = 4.626, *p* = 0.014). As shown in Fig. 3a, scopolamine per se-treated mice significantly reduced hippocampal ACh levels in the hippocampus relative to vehicle controls (*p* < 0.01). Treatment with TCE (200 mg/kg) induced a significant increase in ACh levels when compared with the VEH + SCO group (*p* < 0.05). Donepezil showed a significant increase in ACh levels relative to vehicle controls (*p* < 0.05); this effect was absent when vehicle controls were compared with the TCE per se treated mice compared to vehicle control (Fig. 3a).

We also measured hippocampal AChE activity to elucidate the underlying mechanism of a possible TCE-induced increase of ACh levels. One-way ANOVA indicated a statistically significant between-group effects (F_(5,29)_ = 10.999, *p* < 0.001). In the scopolamine-injected group, a significant increase in AChE activity was observed in the hippocampus (Fig. 3b; *p* < 0.001). TCE administration inhibited AChE activation induced by scopolamine treatment in a dose-dependent manner (Fig. 3b; *p* < 0.01 for 100 mg/kg and *p* < 0.001 for 200 mg/kg). Donepezil also resulted in significant inhibition of AChE activity (*p* < 0.001). The difference in AChE levels between the TCE per se treatment and vehicle treatment were nonsignificant.

**Fig. 4:**
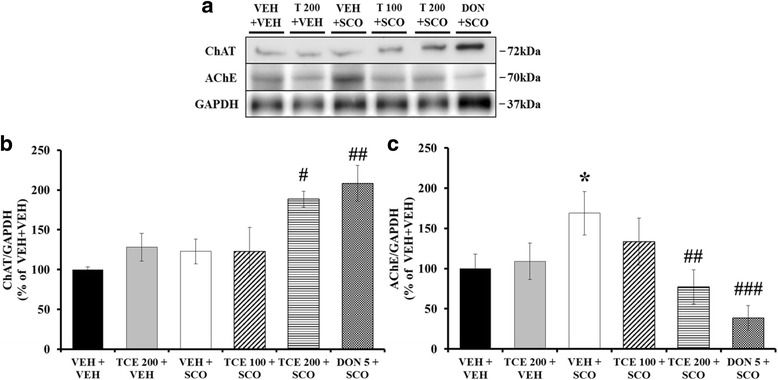
Effect of TCE on hippocampal ChAT and AChE expression in the scopolamine-induced model. **a** Representative protein bands of ChAT and AChE. GAPDH was used as a loading control. **b** The quantification of ChAT expression is shown (*n* = 5 per group). **c** The quantification of AChE expression is shown (*n* = 5 per group). Bar graph is expressed as a percentage of VEH + VEH control. The data are expressed as the means ± S.E.M. *p < 0.05 compared with VEH + VEH; #*p* < 0.05, ##*p* < 0.01, ###*p* < 0.001 compared with VEH + SCO. VEH; vehicle, SCO; scopolamine, T; TCE, DON; donepezil

To examine whether TCE treatment influenced protein expression associated with the cholinergic pathway, we performed western blotting for ChAT and AChE on hippocampal tissue (Fig. 4a). One-way ANOVA revealed between-group effects (F _(5,29)_ = 5.276, *p* = 0.002) in ChAT expression. Consistent with a previous report [16], there were no significant differences between vehicle control and scopolamine per se-treated mice (Fig. 4b). Treatment with scopolamine and TCE at a dose of 200 mg/kg significantly up-regulated the expression of ChAT in the hippocampus (Fig. 4b; *p* < 0.05). Donepezil also showed significantly increased ChAT expression (*p* < 0.01). Moreover, one-way ANOVA indicated between-group changes in the AChE expression levels in the hippocampus (F _(5,29)_ = 3.882, *p* = 0.010): TCE at 200 mg/kg reversed the scopolamine-mediated increase of AChE (Fig. 4c; *p* < 0.01) and Donepezil induced a significant decrease in AChE expression (*p* < 0.001). TCE per se treatment did not exhibit any significant changes when compared with vehicle treatment alone. These findings suggest that TCE may protect against scopolamine-induced memory impairment through mechanisms related to cholinergic function.

### Effect of TCE on oxidative damage in the hippocampus

**Fig. 5:**
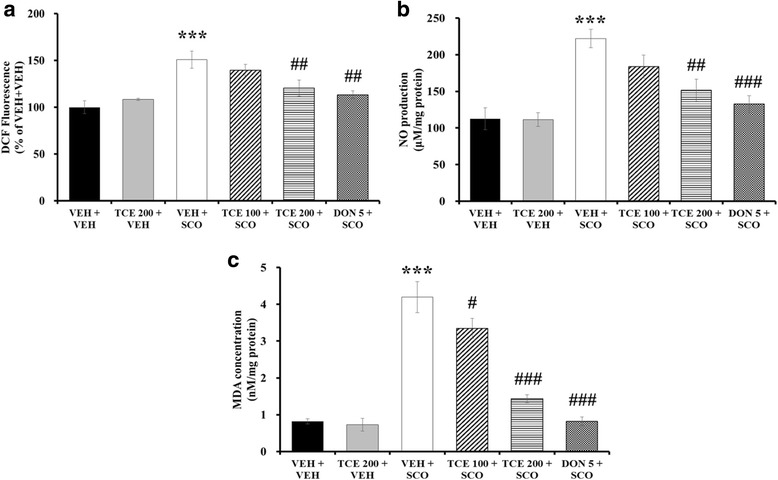
Effect of TCE on oxidative damage induced by scopolamine in the hippocampus of mice. **a** DCF fluorescence in the hippocampus was determined to assess effect of TCE on ROS production (*n* = 3 per group). Bar graph is expressed as the percentage of VEH + VEH control. **b** NO levels in the hippocampus are shown (*n* = 3 per group). **c** MDA concentration in the hippocampus is determined to assess the effect of TCE on lipid peroxidation (*n* = 3 per group). The data are expressed as the means ± S.E.M. ***p < 0.001 compared with VEH + VEH; #*p* < 0.05, ##*p* < 0.01, ###*p* < 0.001 compared with VEH + SCO. VEH; vehicle, SCO; scopolamine, DON; donepezil

We investigated ROS, NO, and MDA levels in hippocampal tissue to determine whether TCE inhibits oxidative damage induced by scopolamine. Regarding the effect of TCE on the ROS production, one-way ANOVA revealed significant between-group effects (F_(5,17)_ = 8.592, *p* < 0.001). The ROS level was significantly increased in scopolamine-treated mice relative to vehicle-treated mice (*p* < 0.001); this elevation was significantly attenuated after treatment with either TCE at 200 mg/kg or donepezil (Fig. 5a; *p* < 0.01). One-way ANOVA revealed significant between-group effects on NO production (F_(5,17)_ = 10.631, *p* < 0.001). Scopolamine administration notably up-regulated NO levels in the hippocampus (*p* < 0.001). Administration of TCE at 100 mg/kg induced a nonsignificant attenuation of NO levels, while the 200 mg/kg dosage induced a significant mitigation (Fig. 5b; *p* < 0.01). Donepezil also resulted in the significant reduction of NO concentration (*p* < 0.001). MDA levels in the hippocampus were also measured to determine the effect of TCE on lipid peroxidation; One-way ANOVA indicated significant changes between groups (F_(5,17)_ = 43.445, *p* < 0.001). Compared with the vehicle treatment group, scopolamine treatment generated a significant increase in MDA concentrations (*p* < 0.001). TCE treatment (100 and 200 mg/kg) affected a significant decrease of MDA levels relative to scopolamine treatment (Fig. 5c; *p* < 0.05 and *p* < 0.001, respectively). A reduced MDA concentration was also observed in the donepezil treatment group (*p* < 0.001). These results suggest that TCE effectively attenuated oxidative damage induced by scopolamine.

### HPLC analysis of TCE

**Fig. 6:**
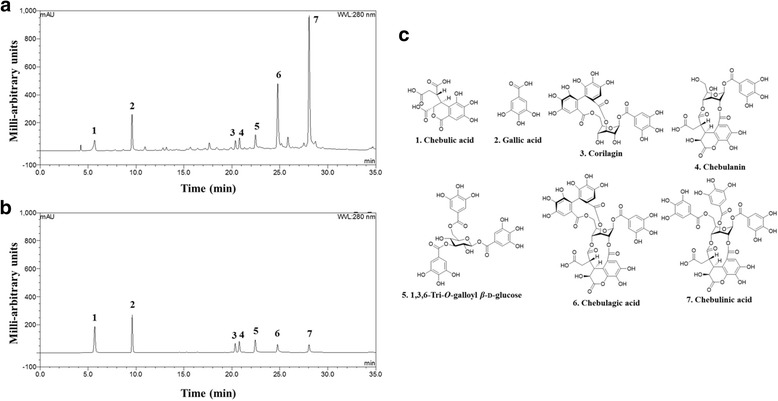
Representative HPLC-UV chromatogram**. a** HPLC-UV chromatogram of TCE. **b** HPLC-UV chromatogram of seven standard compounds in the mixed solution. **c** Chemical structures of the seven main compounds in TCE

The HPLC chromatogram characteristics of the seven compounds in TCE (chebulic acid, gallic acid, corilagin, chebulanin, 1,3,6-tri-O-galloyl β-D-glucose, chebulagic acid, and chebulinic acid), including the retention time and UV spectrum, were determined. The retention times for the seven standards of chebulic acid, gallic acid, corilagin, chebulanin, 1,3,6-tri-O-galloyl β-D-glucose, chebulagic acid, and chebulinic acid were 5.69, 9.60, 20.34, 20.76, 22.43, 24.75, and 28.04 min, respectively (Fig. 6 and Table 1). All of the calibration curves for the seven compounds exhibited good linearity (r^2^ > 0.9990). Table 1 shows that chebulagic acid (169.7 mg/g) and chebulinic acid (263.7 mg/g) are the principal components of TCE.

**Table 1:** Proportion of the seven TCE constituent compounds ^a^

No.	Analytes	Content (%)
1	Chebulic acid	1.60
2	Gallic acid	2.85
3	Corilagin	2.76
4	Chebulanin	2.56
5	1,3,6-Tri-*O*-galloyl *β*-D-glucose	2.82
6	Chebulagic acid	16.97
7	Chebulinic acid	26.37

^a^All analyses were conducted in triplicate

## Discussion

*Terminalia chebula*, the main material examined in the present study, is widely used as a medicinal herb to treat diabetes, asthma, sore throat, and other conditions [20]. Much research has already been conducted to investigate its activity and applications; however, the anti-AChE and anti-oxidative effects of *Terminalia chebula* have primarily been observed in vitro. Though one study previously reported nootropic effects of TCE in normal mice in vivo [21], whether TCE could alleviate amnesia following cholinergic blockade remains unknown. The present study therefore examine the anti-amnesic effect of TCE using mice treated with scopolamine, an inhibitor of central cholinergic transmission. Based on findings from previous reports, we administered TCE at 100 and 200 mg/kg doses [15, 21]. As confirmed by the results the water maze trials, the findings demonstrate that TCE has an anti-amnesic effect on scopolamine-treated mice. We also observed the anti-AChE and anti-oxidative effects of TCE using several assays, including ex vivo experiments. In this respect, *Terminalia chebula* could potentially represent an important alternative drug for addressing amnesia.

In the present study, we confirmed that TCE is composed of seven compounds and ascertained their quantitative differences through an HPLC analysis. Most of the compounds found in TCE are phenolic, including hydrolyzable tannins. We recently reported that chebulic acid and chebulanin showed AChE inhibitory activity at a concentration of 10 μM. In particular, chebulanin showed the most potent AChE inhibitory activity, with an IC_50_ of 21.36 μM [22]. Considering the anti-oxidative effects of both compounds as reported in previous studies [23, 24], chebulanin and chebulic acid may be the major anti-amnesic constituents of TCE. Future in vivo studies are required to determine the utility of these compounds in developing pharmacological agents.

ACh signaling plays a crucial role in maintaining memory function [25]. Since patients with memory disorders typically exhibit cholinergic deficits (i.e., abnormally elevated AChE activity and reduced ACh levels), many studies have focused on the restoration of the cholinergic systems to improve memory [26]. According to a previous report, scopolamine causes memory impairments by means of affecting cholinergic pathways [27]; the scopolamine model used in the present study is therefore suitable for assessing the effectiveness of anti-amnesic agents.

In the present investigation, all mice underwent analysis using the Morris water maze to evaluate learning and memory function. The water maze serves as a powerful and sensitive tool for assessing hippocampal-dependent spatial learning and memory [28]. We conducted a cumulative search error indicating the distance of each mouse from the platform during daily training sessions to characterize spatial learning,; the values thus reflect both proximity and latency [17]. The results demonstrated that TCE treatment improved search error during training trials, suggesting the amelioration of learning impairment. Moreover, the probe test revealed that TCE administration at a dose of 200 mg/kg elevated the time spent in the target quadrant; the results thus evince the efficacy of TCE in improving memory retention. Further study is needed to investigate whether TCE might affect the retrieval of memory, another stage of the memory process.

Our biochemical analyses further demonstrated that TCE exhibits therapeutic effects on cholinergic dysfunction in the hippocampus: a major region of the brain involved in learning and memory function, particularly in spatial memory [29]. Many pathological changes to brain structure and function have been detected in the hippocampus of patients with amnesia; specifically, abnormalities in the cholinergic system were observed in the hippocampus of amnesia patients [26]. It has also been reported that the hippocampus is vulnerable to damage after scopolamine administration [30]. In this context, TCE administration mitigated AChE activity and expression in hippocampal tissue. We also found a negative correlation between AChE activity and the time spent in the target quadrant (Additional file 1: Figure S1). Although ChAT expression was not affected by scopolamine administration, increased ChAT expression was observed in the scopolamine + TCE (200 mg/kg) group. We did not, however, observe significant differences in hippocampal ChAT expression between the vehicle control and TCE per se-treated mice. Further studies are required to reveal the cause of these differences or lack thereof. Taken together, these results suggest that the regulation of the cholinergic system may underlie the anti-amnesic effects of TCE.

Increase in oxidative stress with age is the main risk factor for amnesia; the excessive generation of oxidative products, including ROS, contributes to the disturbance of calcium homeostasis, increased neurotoxic activity, and subsequent elevated lipid peroxidation [31]. Delaying or preventing oxidative damage using anti-oxidants may therefore present a promising therapeutic strategy for treating amnesia. We investigated the anti-oxidative effects of TCE using an in vivo scopolamine model as this model encompasses notable oxidative damage. In our study, increased ROS and MDA concentrations were correlated with memory impairment (Additional file 1: Figure S1). The results showed that TCE treatment attenuated up-regulated levels of ROS, NO, and MDA induced by scopolamine in the hippocampal region, which is vulnerable to oxidative stress [32]. TCE treatment may thus alleviate amnesic symptoms by exerting anti-oxidative effects.

A previous study has reported that levels of AChE splice variants were increased under conditions of oxidative-stress [33]. We therefore analyzed the correlation between AChE activity and oxidative markers and observed a significant correlation between AChE activity and ROS, as well as between AChE activity and MDA (Additional file 2: Figure S2); our findings agree with those of a previous study [34]. These results therefore suggest a possible interaction between scopolamine-induced cholinergic modulation and oxidative stress. However, further research is needed to clarify the mechanism underlying the interaction.

Our study has several limitations. First, we restricted our investigation to spatial learning and memory. The effect of TCE on other types of learning and memory (e.g., object recognition memory and fear memory) remains unknown. Second, we did not investigate which channel(s) or receptor(s) are involved in cholinergic transmission after the administration of TCE. Lastly, we do not know whether TCE has a beneficial effect on amnesia in chronic neurodegenerative diseases, such as Alzheimer’s disease (AD). Further studies are therefore necessary to explore the anti-amnesic effects of TCE using a transgenic animal model of AD.

## Conclusion

The present study was conducted to evaluate the anti-amnesic activity of TCE in a scopolamine-induced murine model of memory impairment. Our data demonstrated that TCE administration ameliorated cognitive deficits measured by performance in water maze tasks. TCE treatment reduced AChE and increased ChAT expression, thus elevating ACh levels in hippocampal tissue of scopolamine-induced mice. Furthermore, decreases ROS, NO, and MDA levels in the hippocampus evince the anti-oxidative capability of TCE treatment. The results indicate that the underlying mechanism of learning and memory improvement may involve modulations of the cholinergic system and the reduction of oxidative stress. These findings thus provide evidence for the potential of TCE extract as a natural, alternative treatment for amnesia.

## Additional files

Additional file 1: **Figure S1.** Correlation graph between behavior test and measured biomarkers. (A) Correlation of time in target quadrant of the first probe test (%) with AChE activity (% of VEH + VEH). (B) Correlation of time in target quadrant of the first probe test (%) with DCF fluorescence (% of VEH + VEH). (C) Correlation of time in target quadrant of the first probe test (%) with MDA concentration (nM/mg protein). (TIF 1767 kb)

Additional file 2: **Figure S2.** Correlation graph between AChE activity and oxidative markers. (A) Correlation of AChE activity (% of VEH + VEH) with DCF fluorescence (% of VEH + VEH). (B) Correlation of AChE activity (% of VEH + VEH) with MDA concentration (nM/mg protein). (TIF 1099 kb)

## Abbreviations

ACh: Acetylcholine
AChE: Acetylcholinesterase
AD: Alzheimer’s disease
ChAT: Choline acetyltransferase
DCFDA: Dichlorofluorescin diacetate
DMSO: Dimethyl sulfoxide
GAPDH: Glyceraldehyde 3-phosphate dehydrogenase
HPLC: High-performance liquid chromatography
MDA: Malondialdehyde
NO: Nitric oxide
ROS: Reactive oxygen species
TCE: *Terminalia chebula* extract
