# Supplementary figures and images for: Terminalia chebula extract prevents scopolamine-induced amnesia via cholinergic modulation and anti-oxidative effects in mice
Source: BMC Complement Altern Med. 2018 May 2;18:136. doi: 10.1186/s12906-018-2212-y (PMC5930767; doi:10.1186/s12906-018-2212-y)

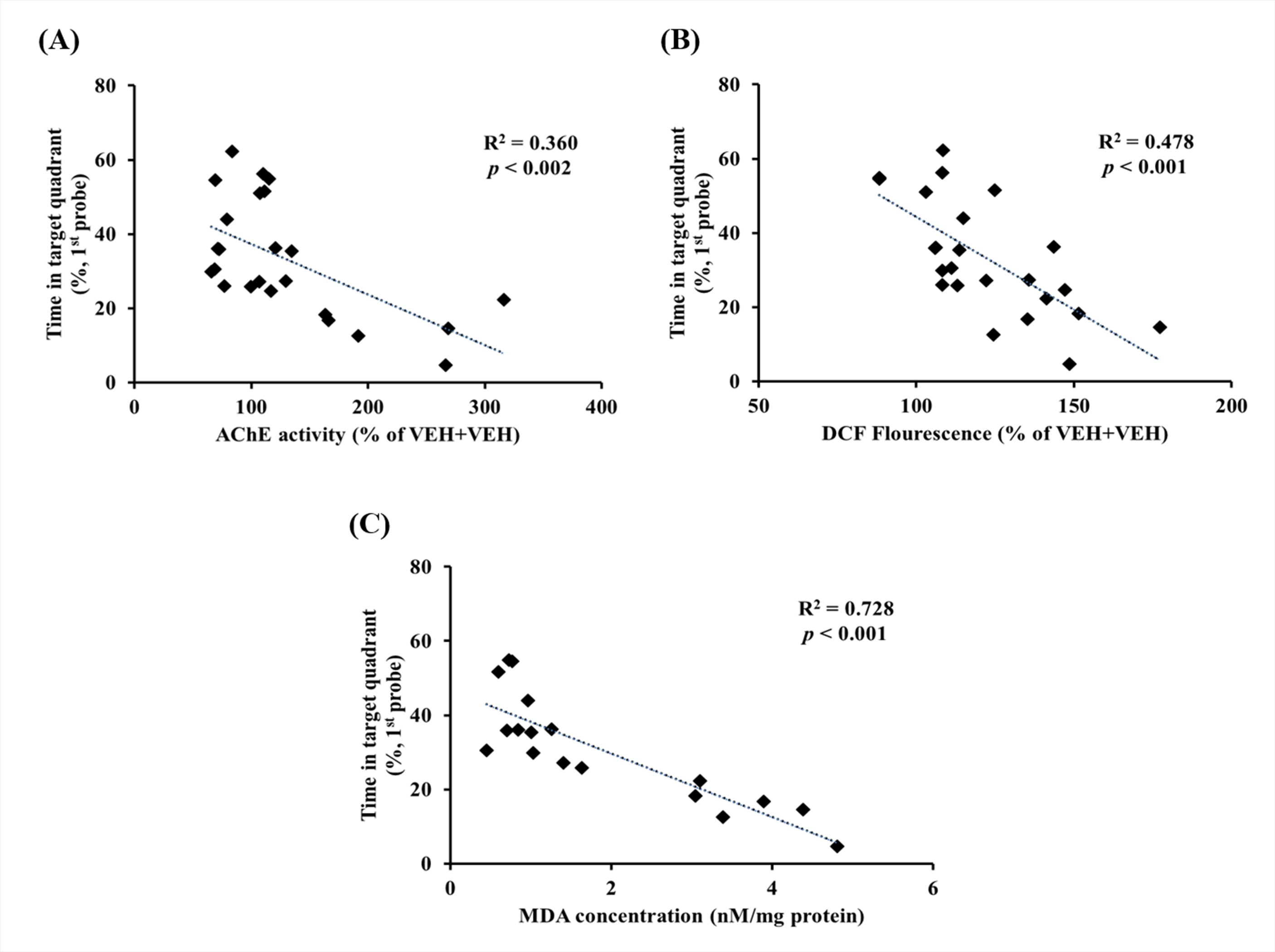

Supplement: Supplementary file 1 — Figure S1. Correlation graph between behavior test and measured biomarkers. (A) Correlation of time in target quadrant of the first probe test (%) with AChE activity (% of VEH + VEH). (B) Correlation of time in target quadrant of the first probe test (%) with DCF fluorescence (% of VEH + VEH). (C) Correlation of time in target quadrant of the first probe test (%) with MDA concentration (nM/mg protein). (TIF 1767 kb) [file 12906_2018_2212_MOESM1_ESM.tif]

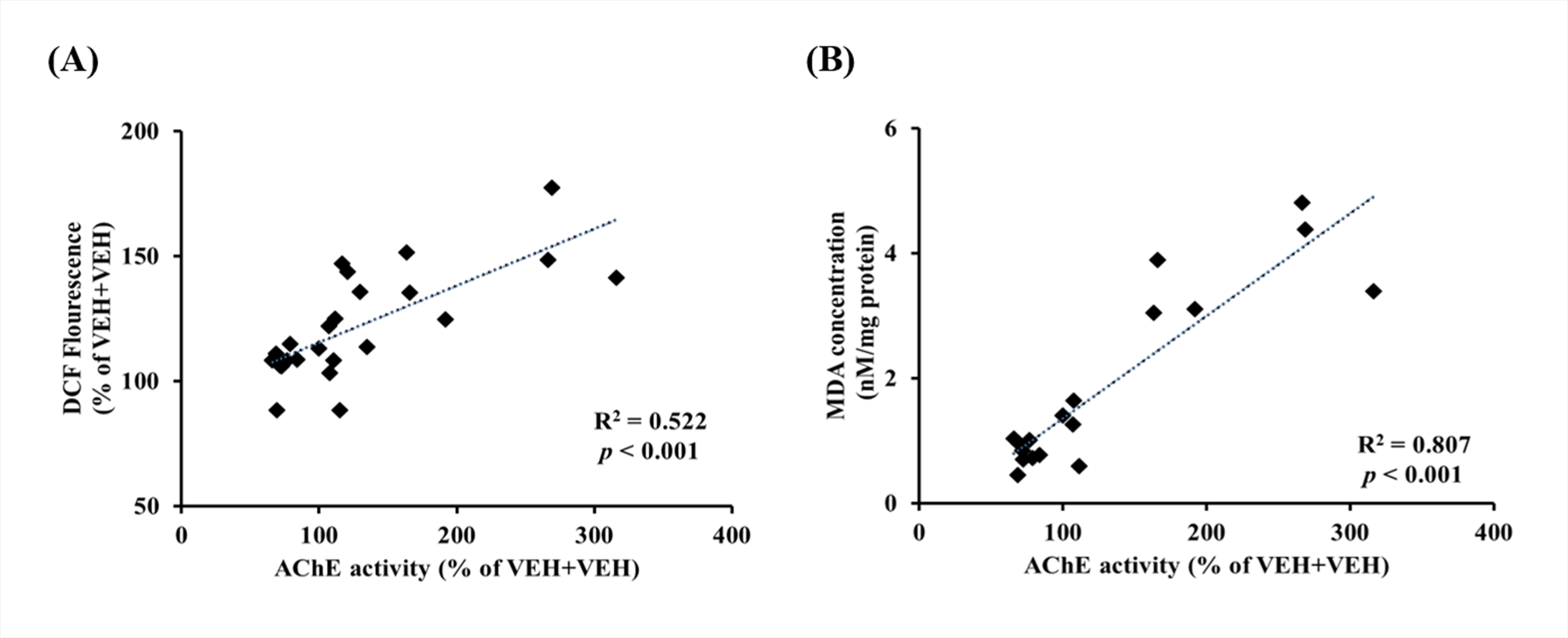

Supplement: Supplementary file 2 — Figure S2. Correlation graph between AChE activity and oxidative markers. (A) Correlation of AChE activity (% of VEH + VEH) with DCF fluorescence (% of VEH + VEH). (B) Correlation of AChE activity (% of VEH + VEH) with MDA concentration (nM/mg protein). (TIF 1099 kb) [file 12906_2018_2212_MOESM2_ESM.tif]
